# Freshwater Aquaculture Nurseries and Infection of Fish with Zoonotic Trematodes, Vietnam

**DOI:** 10.3201/eid1612.100422

**Published:** 2010-12

**Authors:** Van Thi Phan, Annette Kjær Ersbøll, Thanh Thi Nguyen, Khue Viet Nguyen, Ha Thi Nguyen, Darwin Murrell, Anders Dalsgaard

**Affiliations:** Author affiliations: Research Institute for Aquaculture No.1, Bac Ninh, Vietnam (V.T. Phan, K.V. Nguyen, H.T. Nguyen);; University of Southern Denmark, Odense, Denmark (A.K. Ersbøll);; Vinh University, Vinh, Vietnam (T.T. Nguyen);; University of Copenhagen, Copenhagen, Denmark; (V.T. Phan, D. Murrell, A. Dalsgaard)

**Keywords:** Foodborne infections, parasites, zoonoses, aquaculture, trematodes, juvenile fish, fry, hatchery, Vietnam, research

## Abstract

Residents of the Red River Delta region of northern Vietnam have a long tradition of eating raw fish. Fish-borne zoonotic trematodes (FZTs) are estimated to infect ≈1 million persons in Vietnam. It remains uncertain at what stages in the aquaculture production cycle fish become infected with FZTs. Newly hatched fish (fry) from 8 hatcheries and juveniles from 27 nurseries were therefore examined for FZT infection. No FZTs were found in fry from hatcheries. In nurseries, FZT prevalence in juveniles was 14.1%, 48.6%, and 57.8% after 1 week, 4 weeks, and when overwintered in ponds, respectively. FZT prevalence was higher in grass carp (p<0.001) than in other carp species. Results show that nurseries are hot spots for FZT infections in fish. Thus, sustainable FZT prevention strategies must address aquaculture management practices, particularly in nurseries, to minimize the risk of distributing infected juveniles to grow-out ponds and, subsequently, to markets for human consumption.

## Abstract 2

TOC summary: Aquaculture nurseries should be included in control programs to produce parasite-free fish for human consumption.

Liver and intestinal infections caused by fish-borne zoonotic trematodes (FZTs) are increasingly being recognized as serious public health problems and with FZTs incorporated among causes of neglected tropical diseases (*1**,**2*). FZTs are especially widespread in Southeast Asia, including Vietnam, Lao People’s Democratic Republic, Thailand, Cambodia, People’s Republic of China, and North and South Korea (*3**–**9*). Liver flukes are associated with high incidence of bile duct cancer (*1**,**10*), and intestinal flukes cause serious pathologic changes in the heart, brain, and spinal cord (*1**,**2**,**11*).

The epidemiology of FZTs is complex because humans and reservoir hosts, such as dogs, cats, pigs, and fish-eating birds, harbor egg-shedding adult stages (*12**,**13*). These hosts are infected by consumption of raw, inadequately cooked, or pickled fish. For many inhabitants in the Red River Delta provinces of northern Vietnam, the consumption of such fish dishes is a traditional behavior that is difficult to alter (*14**–**16*). In the Nam Dinh and Ninh Binh provinces, the widespread habit of eating raw fish is associated with a high FZT prevalence of 30%–40% in humans (*3**,**4*). Aquaculture fish species commonly used to prepare raw fish dishes, such as carp, frequently also have high a prevalence of FZT metacercariae (*12**,**17**–**19*).

The influence of FZTs on the food safety of aquaculture products can have a noticeable adverse economic and public health effect because fish farming in Asia is expanding rapidly. Farm-raised fish are a main protein source consumed domestically and an essential product for exporting to other countries (*2**,**10**,**20*). Therefore, the production of FZT-free fish for human consumption should be a key objective for the aquaculture industry. Achieving this goal is seriously hampered, however, because the present state of knowledge on FZT infection in the fish production chain is inadequate to devise practical and sustainable prevention strategies, especially for small-scale and integrated freshwater aquaculture. The available knowledge of FZT infection is mainly obtained from studies of fish in grow-out ponds, where fish are harvested for human consumption (*12**,**17**,**19*).

Freshwater fish hatcheries in Nam Dinh, Ninh Binh, and Bac Ninh provinces include facilities such as a water reservoir, water storage facilities, breeding tanks, and incubators for hatching eggs. Depending on the hatchery, the water reservoirs are cement tanks or consist of earthen ponds from which the water is either pumped into cement breeding tanks or supplied from a tower to the breeding tanks and egg incubators. Water used for the breeding tanks and incubators is filtered through a net to remove different microbiota, e.g., zooplankton. Brood stock are moved from earthen ponds into cement breeding tanks for induced spawning. Fertilized eggs are then incubated in round cement incubators with running water for ≈5 days, depending on fish species and temperature. Newly hatched fish are termed fry. Fry are kept in tanks for 3–5 days, after which they are sold and subsequently stocked in earthen ponds in so-called nurseries.

The fish raised in nursery ponds are called juveniles. The nursery ponds in Nam Dinh and Ninh Binh provinces are mainly backyard earthen ponds located close to households and premises housing livestock and poultry. Juveniles are nursed up to 4 weeks and then sold for further nursing to bigger size or to be stocked to reach market size in grow-out ponds. Juveniles may be kept in ponds during the winter months (overwintered juveniles) for sale in early spring.

Management of nurseries in northern Vietnam often involves the application of livestock manure as fertilizer before stocking fish to increase the density of plankton that serves as a food source for juveniles. Additionally, farmers may also apply night soil (human manure) as fertilizers to the nursing ponds.

We report an investigation that aimed to determine the FZT infection status in integrated small-scale hatcheries and nurseries in Nam Dinh, Ninh Binh, and Bac Ninh provinces, which are major areas endemic for FZTs in Vietnam. By assessing the FZT metacercariae prevalence in fish from the initial stages of production, namely the hatcheries and nurseries, the study provides knowledge needed for a comprehensive assessment of FZT infection during the entire fish production cycle.

## Material and Methods

### Fish Sampling and Examinations

In a cross-sectional survey, fry were sampled from 3 hatcheries in Nam Dinh Province, 1 hatchery in Ninh Binh Province, and 4 hatcheries in Bac Ninh Province. In each hatchery, 500 fry of individual fish species, including grass carp (*Ctenopharyngodon idellus)*, silver carp (*Hypophthalmichthys molitrix*), rohu (*Labeo rohita*), mrigal (*Cirrhinus mrigala*), and common carp (*Cyprinus carpio*) were sampled by using a scoop net.

Juveniles were collected from 14 nurseries in Nam Dinh and 13 nurseries in Ninh Binh provinces by cast net (4-week old and overwintered juveniles) or scoop (1-week-old juveniles) nets. Fish were sampled twice from the nursing ponds, initially 1 week after the fry had been stocked and a second time at the end of the 1-month nursing period. In addition, overwintered juveniles were sampled from the same nurseries to compare their infection status with juveniles sampled before the winter period. On the basis of sampling size calculations, 15 juveniles were collected for each species at each time of sampling in each nursery.

The sample fish were transported to the laboratory for metacercariae examination and kept alive in plastic bags with added oxygen. The length and weight of each fish was recorded before samples were processed.

FZT infections in fry were examined by placing 5 fry on a glass slide, compressing them with another slide, and then examining for trematodes under a stereo microscope (×4) and a compound microscope (×100). Juveniles were digested in 1% pepsin to release metacercariae, following the procedure described by the World Health Organization (*1*) and modified by Chi et al. (*12*). Identification of the metacercariae was made according to morphologic features detailed by Pearson and Ow-Yang (*21*), Scholtz et al. (*22*), and Kaewkes (*23*).

### Data Analysis

SAS version 9.1 (SAS Institute, Inc., Cary, NC, USA) was used for statistical analysis with fish as the study unit. Descriptive analyses of FZT infection in fry and juveniles were conducted by frequency distribution. Density of metacercariae in 1-week-old juveniles was calculated as number of metacercariae per whole fish, and density of metacercariae in 4-week-old juveniles and overwintered juveniles was calculated as number of metacercariae per gram of fish. Because of a skewed distribution of the density of metacercariae in fish, descriptive analysis was performed by means of median, Q_1_ (25% percentile), Q_3_ (75% percentile), minimum values, and maximum values.

Logistic regression analysis was used to evaluate prevalence differences of FZT between provinces, nurseries systems and fish species. A p value <0.05 was considered significant.

## Results

### FZT Species

No FZT metacercariae were found in fish fry from hatcheries in Nam Dinh, Ninh Binh, and Bac Ninh provinces. Table 1 shows the FZT species recovered from the various fish species and nursery systems examined in Nam Dinh and Ninh Binh provinces. The liver fluke *Clonorchis sinensis* was found in only 1.5% of fish. Intestinal flukes, including *Haplorchis pumilio* and *Centrocestus formosanus*, were found in 55.6% and 41.0%, respectively, of the total number of FZT-infected juveniles. Several FZT species were found in individual FZT-infected juveniles.

**Table 1:** Descriptive analysis of fishborne zoonotic trematode and fish species in nursery systems in Nam Dinh and Ninh Binh provinces, northern Vietnam*

Fish species and culture system	Fishborne zoonotic trematode species, n/N (%)			
*Clonorchis sinensis*	*Haplorchis pumilio*	*Haplorchis taichui*	*Centrocestus formosanus*
Overall	12/797 (1.5)	443/797 (55.6)	2/797 (0.3)	327/797 (41.0)
Fish				
Grass carp	6/313 (1.9)	213/313 (68.1)	1/313 (0.3)	186/313 (59.4)
Rohu	1/208 (0.5)	92/208 (44.2)	–	65/208 (31.3)
Mrigal	0/55 (0)	17/55 (30.9)	–	2/55 (3.6)
Pacu	0/8 (0)	6/8 (75.0)	–	5/8 (62.5)
Silver carp	5/213 (2.4)	115/213 (53.99)	1/313 (0.3)	69/213 (32.4)
Culture system				
1-week-old juveniles	0/197 (0)	87/197 (44.2)	–	138/197 (70.1)
4-week-old juveniles	5/277 (1.8)	224/277 (80.9)	1/277 (0.4)	133/277 (48.0)
Overwintered juveniles	7/323 (2.2)	132/323 (40.9)	1/323 (0.3)	56/323 (17.3)

*n, no. fish infected with specific fishborne zoonotic trematode species; N, no. fishborne zoonotic trematode–infected fish; –, no parasites found.

### FZT Prevalence in Juvenile Fish

Table 2 shows the prevalence of FZTs in fish juveniles from Nam Dinh and Ninh Binh provinces and in different age groups of juveniles. Juveniles were already infected (14.1%) during the first week of exposure in the nursery ponds with a further significant increase (48.6%) after 4 weeks nursing when the juveniles were either sold for further stocking or kept in ponds during the winter months. Some additional but nonsignificant infection took place during the 5–6-month overwintering period.

**Table 2:** Fishborne zoonotic trematode infections among juvenile fish in nurseries, northern Vietnam*

Province and fish age	No. FZT-infected/total no. (%)
Overall	797/2,524 (31.6)
Province	
Nam Dinh	448/1,761 (25.4)
Ninh Binh	349/763 (45.7)
Fish age	
1-week-old juveniles	197/1,395 (14.1)
4-week-old juveniles	277/570 (48.6)
Overwintered juveniles	323/559 (57.8)

*FZT, fishborne zoonotic trematode.

### FZT Density in Juvenile Fish

Among infected 1-week-old juveniles, 50% contained only 1 metacercaria/fish, 25% contained 2–4 metacercariae/fish, and 25% contained >5–18 metacercariae/fish. Among the 4-week-old juvenile fish, 50% had metacercariae densities <1.7/g fish tissue, 25% had densities of 1.7–<6.7/g fish tissue, and 25% had <6.7–173.3/g of fish tissue. In overwintered juveniles, 50% had a density <0.8/g, 25% had a density of 0.8–<2.3/g, and 25% had a density of 2.3–170.2/g. An explanation for this apparent decrease in density over the winter is that when overwintered juveniles have the same density as the non-overwintered juveniles at the start of the overwintering period, then as the fish grows during the overwinter period, the density will decrease if FZT transmission is reduced during the colder winter. The significant increase in FZT prevalence seen among overwintered juveniles compared with 4-week-old juveniles indicates that some transmission of FZTs occurs during the winter.

Table 3 shows the result of logistic analysis of FZT prevalence in 5 fish species and 3 nursery systems in Nam Dinh and Ninh Binh provinces. Fish juveniles from Ninh Binh Province had significantly higher risk for being infected with FZT than those from Nam Dinh Province (p = 0.012). The odds of FZT infection for grass carp was 6× higher than for Rohu (p<0.0001), and silver carp had a 1.3× higher risk for FZT infection than Rohu. Combined for all fish species, overwintered juveniles and juveniles cultured for 4 weeks had odds of FZT infection that were 12.9× and 3.3× higher, respectively, than 1-week-old nursed fry. The nursing system had a significant effect (p<0.0001) on FZT prevalence in 1-week-old juveniles.

**Table 3:** Logistic regression analyses of fishborne zoonotic trematode prevalence found in different fish species and nursery systems in Nam Dinh and Ninh Binh provinces, northern Vietnam*

Variable	Estimate†	SE	OR (95% CI)	p value
Intercept	−1.672	0.2877		
Province				0.0123
Nam Dinh	1.026	0.3803	0.359 (0.164–0.785)	
Ninh Binh‡	0		1	
Fish				<0.0001
Grass carp	1.794	0.1668	6.013 (4.336–8.340)	
Silver carp	0.284	0.1558	1.329 (0.979–1.804)	
Mrigal	0.032	0.2260	1.033 (0.663–1.609)	
Rohu‡	0		1	
Culture system				<0.0001
1-week-old juveniles‡	0		1	
4-week-old juveniles	1.194	0.1485	3.299 (2.465–4.414)	
Overwintered juveniles	2.552	0.1684	12.831 (9.222–17.852)	

*OR, odds ratio; CI, confidence interval. †Parameter estimate of variables in the resulting model. ‡Referent.

## Discussion

The finding in this study that fish become infected with FZT as early as in the first week of growth in the nurseries and have a 48.6% infection prevalence when 4 weeks of age is of great consequence for public health and the aquaculture industry. It indicates that infection in nursery juveniles may account for most of the FZT prevalence reported for fish from grow-out ponds (*12**,**17*). Furthermore, because juveniles produced at nurseries are sold and distributed to numerous grow-out farms, the risk for FZT infection becomes widely distributed throughout the fish farming areas, complicating efforts to control this zoonosis. For example, the acquisition of FZT infection by overwintered juveniles in Nam Dinh and Ninh Binh provinces has implications for grow-out farms in nearby provinces such as Thanh Hoa, Thai Binh, and Ha Nam, as well as in provinces further north, which are major customers for these fish.

The increase of FZT prevalence during the first month in the nursing ponds is much higher than that during the ≈12-month grow-out period in Nam Dinh Province (*19*). Among possible explanations for this finding are more risky management practices by nursery operators or biological factors such as age-related fish susceptibility, resistance factors, and immunity. Further studies are urgently needed to understand the causes for this higher prevalence during the nursing phase and to develop efficient prevention management.

Grass carp, one of the favored sources of raw fish for human consumption, had a significantly higher FZT prevalence than other fish species, which is consistent with results of a previous study (*24*). This may be due to the carp’s food preference for grass and aquatic plants. In these nursing ponds, the grass and aquatic plants used for feeding were often harvested outside the farm, which may have introduced FZT-infected snails into the ponds. According to Phan et al. (*19*), feeding with green vegetation collected outside the farm is a risk factor for FZT infection in cultured fish. Another factor may be that grass carp spend more time in the littoral zone where vegetation and therefore snails may be concentrated. Furthermore, grass carp might be more susceptible to FZT infection than other fish species.

The higher prevalence of FZT metacercariae in Ninh Binh Province may be due to the common practice of using night soil to fertilize ponds to increase growth of plankton, a major source of carp feed. Nam Dinh Province also has a high human and domestic animal FZT prevalence (*3**,**4**,**13*), making fecal waste from these hosts a highly risky fertilizer. To develop effective control interventions, more research on methods to inactivate FZT eggs in manure must be conducted.

The lack of infection in fish fry is consistent with the report of Thien et al. (*24*), who investigated fry from hatcheries in the Mekong Delta. Fry were raised in well-controlled hatcheries by using tanks or ponds containing filtered water free of zooplankton, snails, and other microbiota.

One limitation of our study is that bias could occur when sampling fish by using 2 types of net, i.e., cast net and scoop net. However, cast nets cannot be used in fry incubators in hatcheries or to catch 1-week-old juveniles. Different methods for examining and isolating FZT metacercariae in fish fry and juveniles are needed because compression of whole fish bodies is efficient for processing of small fry and detecting any metacercariae present. For larger fish such as juveniles, however, the thickness of the body and muscle tissue precludes using compression, and instead, pepsin digestion (a more laborious procedure) is the method of choice. We also have compared pepsin digestion for several cups of fry with compressing methods and found no significant difference in detection efficiency.

Liver fluke (*C. sinensis*) metacercariae had a low prevalence (1.5%); most FZT species found were intestinal flukes, particularly *Haplorchis* spp. and *Procerovum varium.* Although generally not considered to have a clinical role compared with liver flukes, several intestinal FZTs can cause serious pathologic effects, even death, when infecting the heart, brain, and spinal cord of humans (*1**,**9**,**11*). The dominance of *H. pumilio* trematodes in juveniles is similar to the findings of other studies of nurseries (*24*) and grow-out systems (*12**,**17**,**18*). *Stellantchasmus falcatus,* however, is a major FZT species found in fish from southern and central Vietnam. Although Trung Dung et al. (*4*) recently recovered *S. falcatus* trematodes from humans in Nam Dinh, this species was not found in fish from this province, possibly because humans acquired their infections from fish originating from provinces other than Nam Dinh.

The life cycle and epidemiology of FZTs in aquaculture systems is complicated, and fish are vulnerable to multiple risks for infection. However, by developing a hazard analysis of critical control points approach to identifying points for intervention and enlisting the collaboration between different sectors such as human health, animal health, snail vector control, and aquaculture management and extension, we believe a major reduction in FZTs in farmed fish can be achieved, with the benefit of being able to provide aquaculture products free of FZTs for human consumption.

In conclusion, the results from this study strongly suggest that a program for the prevention for FZTs in farmed fish must include nurseries and grow-out farms. An integrated program will, however, require more research on the infection events occurring in the nursery pond, particularly the regulation of snail populations, fish risk behavior, and on methods to inactivate FZT eggs in manure intended for fertilizer use.
